# Whole exome sequencing identifies concurrent *LDLR* and *ABCG8* mutations in a Saudi family with familial hypercholesterolemia and Sitosterolaemia

**DOI:** 10.3389/fgene.2025.1679594

**Published:** 2025-10-16

**Authors:** Abdulrahman Hummadi, Dhayf Alrahman Mutawwam, Babajan Banaganapalli, Fai Alsubhi, Naji J. Aljohani, Ali J. Alhagawy, Turki Algohani, Ibrahim Fallatah, Mohammed Y. Daghriry, Rawan K. Alharbi, Hassan Alhafaf, Madi Talal, Rania R. Hassan

**Affiliations:** ^1^ Adult Endocrinology and Diabetes, Jazan Endocrinology & Diabetes Center, Ministry of Health, Jazan, Saudi Arabia; ^2^ Molecular Genetics Department, Jazan Health Affairs, Ministry of Health, Jazan, Saudi Arabia; ^3^ Department of Genetic Medicine, Faculty of Medicine, King Abdulaziz University, Jeddah, Saudi Arabia; ^4^ Princess Al- Jawhara Al-Brahim Center of Excellence in Research of Hereditary Disorders, King Abdulaziz University, Jeddah, Saudi Arabia; ^5^ Department of Pathology and Laboratory Medicine, Ministry of the National Guard-Health Afairs, King Abdulaziz Medical City-WR, Jeddah, Saudi Arabia; ^6^ Consultant Endocrinology, Obesity, endocrine and Metabolic Center, King Fahad Medical City, Riyadh, Saudi Arabia; ^7^ College of Medicine, Alfaisal University, Riyadh, Saudi Arabia; ^8^ Department of Pathology And Laboratory Medicine, King Abdulaziz Medical City-Central Region, Riyadh, Saudi Arabia

**Keywords:** familial hypercholesterolemia, sitosterolemia, LDLR mutation, ABCG8 variant, whole exome sequencing, molecular docking

## Abstract

**Background:**

Sitosterolemia and Familial hypercholesterolemia (FH) represent two genetically distinct lipid metabolism disorders marked by disparate inheritance mechanisms and therapeutic responses. It is typically inherited in an autosomal dominant pattern due to mutations in the *low-density lipoprotein receptor* (*LDLR*) gene, whereas sitosterolemia follows an autosomal recessive mode associated with mutations in the ATP-binding cassette transporters (*ABCG5* and *ABCG8*). To the best of our knowledge, the presence of both disorders within the same family has never been documented in the scientific literature.

**Objective:**

In this paper, we report what is likely the first genetically confirmed case of compound heterozygosity involving both sitosterolemia and familial hypercholesterolemia in a Saudi Arabian consanguineous family. This unique case highlights the complex diagnostic challenges and therapeutic considerations in managing overlapping dyslipidemia phenotypes.

**Methods:**

A multigenerational family was recruited from the Diabetes and Endocrinology Center in Jazan, Saudi Arabia. Comprehensive clinical evaluations were conducted, including family history, physical examination, and lipid profiling. Whole exome sequencing (WES) was performed using the CentoXome^®^ platform with >98% of targeted bases covered at ≥20x, followed by bioinformatics analysis via a standardized pipeline. Sanger sequencing validated the identified variants. Variant pathogenicity was evaluated using *in silico* tools such as SpliceAI, REVEL, MetaLR, and SIFT, alongside conservation and gene expression data. Statistical analysis of lipid levels pre- and post-treatment was conducted using paired t-tests, with significance set at *p* < 0.05. Notably, direct measurements of plant sterols were not performed.

**Findings:**

WES revealed a novel heterozygous frameshift deletion in *LDLR* and a pathogenic splice site variant in *ABCG8*, consistent with compound FH and sitosterolemia. The proband responded remarkably to ezetimibe monotherapy, while his children required combination therapy with high-intensity rosuvastatin and PCSK9 inhibitor Evolocumab for LDL-C reduction. Structural modeling and molecular docking analyses revealed altered ligand-binding affinities in mutant proteins, providing a plausible structural explanation for the observed variation in drug response.

**Conclusion:**

This study presents the first extensive molecular characterization of a dual FH-sitosterolemia phenotype. It emphasizes the critical role of genomic diagnostics in managing complex lipid disorders and supports personalized medicine approaches, especially in consanguineous populations where blended phenotypes may be underrecognized.

## 1 Introduction

The complex relationship among early onset cardiovascular diseases and inherited metabolic disorders represents a significant challenge in current medical standards, particularly among populations with distinctive genetic predispositions. Within this complicated environment of hereditary dyslipidemias, familial hypercholesterolemia (FH)(OMIM 143890) appears as one of the most prevalent monogenic disorders, exhibiting a global incidence of approximately 1:250 for the heterogeneous variant and a substantially rarer 1:160,000-300,000 for the homozygous manifestation (Nordestgaard et al., 2013; Jahn et al., 2022). The molecular underlying causes of FH primarily involve pathogenic mutations in genes encoding vital components of the LDL-cholesterol metabolic pathway, especially LDLR, PCSK9 and APOB leading to characteristic clinical symptoms including persistently increased LDL-cholesterol concentrations, xanthelasma palpebrarum (manifesting as characteristic yellowish cholesterol deposits in the periorbital tissues), distinctive tendinous xanthomatosis (frequently affecting the Achilles tendons and digital extensor tendons), and accelerated atherogenesis culminating in premature coronary artery disease (Wiegman et al., 2015; Cuchel et al., 2015). The diagnostic approach to FH has progressed significantly, including validated clinical algorithms such as Simon Broome register and Dutch Lipid Clinic Network criteria, supplemented by molecular diagnostic techniques and comprehensive family history assessment. However, the main issue is that most FH cases remain clinically unrecognized until the manifestation of overt cardiovascular complications, including myocardial infarction or angina pectoris which lead to thereby delaying the implementation of appropriate therapeutic interventions and significantly elevating the risk of premature mortality (Sturm et al., 2018; Harada-Shiba et al., 2023; Sing et al., 2015). Current management strategies for FH places highest priority on rapid interventions to reduce lipid levels, with high-potency statins constituting the foundation of pharmacological therapy, often enhanced by ezetimibe to promote LDL-cholesterol reduction through complementary mechanisms. In cases characterized by extraordinary severity or insufficient response to conventional therapies, advanced interventions including lipoprotein apheresis or PCSK9 inhibitors may be warranted (Gidding et al., 2015; Raal et al., 2018). In contrast, sitosterolemia represents an extremely rare autosomal recessive disorder, with an estimated prevalence less than 1:1,000,000, However, this number may not reflect its true prevalence due to substantial diagnostic challenges and phenotypic heterogeneity (Tada et al., 2022). The molecular pathogenesis of sitosterolemia results from biallelic mutations affecting the *ABCG5 or ABCG8* genes, these two genes encode ATP-binding cassette transporters critical for regulating intestinal absorption and biliary excretion of plant sterols. Interruption of these transporters leads to accumulation of non-cholesterol sterols, particularly sitosterol and campesterol within plasma and other tissues (Hubacek et al., 2001; Wang et al., 2015). The clinical presentation of sitosterolemia can highly resemble those of FH, involving hypercholesterolemia and xanthomatosis, however sitosterolemia may additionally encompass distinctive hematological abnormalities, including thrombocytopenia and hemolytic anemia, which are not typically associated with FH (Escolà-Gil et al., 2014; Qin et al., 2022). Whole exome sequencing (WES) has revolutionized the detection of rare and complex genetic disorders by enabling comprehensive interrogation of all protein-coding regions of the genome. This approach is particularly powerful in identifying digenic inheritance patterns, where pathogenic variants in two distinct genes contribute jointly to the phenotype—an aspect often missed by targeted gene panels or traditional diagnostic approaches (Goh and Choi, 2012; Kerner et al., 2020). Despite its power, WES generates a large volume of data that includes thousands of variants per individual, posing significant bioinformatics challenges in distinguishing benign polymorphisms from disease-causing mutations. Accurate variant filtration, annotation, and prioritization require robust computational pipelines and integration of multiple data sources (Roy et al., 2018; Magnusson, 2021). To support the clinical interpretation of variants, *in silico* prediction tools such as SpliceAI, SIFT, and REVEL are essential. These tools evaluate the potential impact of sequence alterations on splicing, protein structure, and function, aiding in the classification of variants based on ACMG/AMP guidelines (Ghosh et al., 2017). Definitive diagnosis requires specialized evaluation of plasma plant sterol concentrations coupled with targeted genetic analysis of the ABCG5/8 loci. The main distinction between these disorders lies in their differential therapeutic responsiveness: whereas FH commonly exhibits robust response to statin therapy, sitosterolemia demonstrates minimal improvement with HMG-CoA reductase inhibition but shows remarkable sensitivity to ezetimibe and dietary plant sterol restriction (Escolà-Gil et al., 2014). The clinical overlap between these two diseases often associated with diagnostic challenges (Qin et al., 2022; Wang W. et al., 2018), This situation increase in complexity in populations characterized by high rates of consanguinity because the complex patterns of inheritance including multiple genetic determinants may occur with greater frequency. In this manuscript, we describe what appears to be the first genetically confirmed instance of a Saudi Arabian family harboring concurrent pathogenic variants in both *LDLR* and *ABCG8*, resulting in a compound heterozygous dyslipidemia phenotype with distinctive therapeutic implications. This exceptional case underscores the critical importance of comprehensive genomic characterization in guiding personalized therapeutic strategies and avoiding the potential consequences of diagnostic misclassification and suboptimal clinical management.

## 2 Materials and methods

### 2.1 Recruitment of familial hypercholesterolemia cases and relatives

This study was conducted with full ethical approval from the Institutional Ethical Committee of Jazan Health Cluster, Kingdom of Saudi Arabia, (Approval Number: 2564) in line with globally accepted ethical guidelines for human subjects’ research. We recruited a multi-generational family presenting with clinical symptoms indicative of familial hypercholesterolemia (FH) through the specialized Diabetes and Endocrinology Center in Jazan. The pedigree included four affected individuals extending across two generations, with the index case being a 55-year-old male exhibiting classical FH phenotypic features, involving characteristic tendinous xanthomatosis, markedly elevated LDL-cholesterol concentrations, and a significant family history of early onset cardiovascular morbidity and mortality. Comprehensive clinical and biochemical assessment were performed for the offspring, who demonstrated laboratory parameters consistent with inherited dyslipidemia. All accessible family members underwent thorough clinical assessment, including detailed lipid profile analysis and exhaustive medical history documentation, with particular emphasis on cardiovascular risk factors and manifestations. Genetic analysis was later undertaken to validate the molecular diagnosis and determine the mode of inheritance.

### 2.2 DNA extraction

We collected 5 mL of Peripheral venous blood specimens (in ethylenediaminetetraacetic acid (EDTA)-containing vacutainers from each participating family member by following informed consent procedures. Genomic DNA was isolated utilizing the QIAamp DNA Blood Mini Kit (Qiagen GmbH, Hilden, Germany) in strict accordance with the manufacturer’s standardized protocol. Quantitative and qualitative evaluation of the extracted DNA was conducted by using a NanoDrop™ spectrophotometer (Thermo Fisher Scientific, Waltham, MA, United States), with particular attention to A260/A280 and A260/A230 ratios to assess protein and organic contaminants, respectively. Then, using 1% agarose gel electrophoresis with ethidium bromide staining, DNA integrity was confirmed.

### 2.3 Whole exome sequencing (WES)

Genomic DNA was enzymatically fragmented following the CentoXome^®^ MOx 1.0 Solo protocol (CENTOGENE GmbH, Germany). Library preparation was performed using the CentoXome^®^ Solo kit, which targets approximately 41 Mb of the coding human exome and mitochondrial genome, based on the GRCh37/hg19 reference assembly. Target enrichment utilized hybridization-based capture probes. Sequencing was conducted on an Illumina HiSeq platform, generating paired-end 150 bp reads. Multiple indexed samples were pooled per sequencing lane, with up to 12 samples per run, allowing efficient multiplexing while maintaining high coverage and data quality. Sequencing achieved >99.4% of targeted regions covered at ≥20x, exceeding the protocol’s baseline target of 98%. A minimum of 3 µg of high-quality genomic DNA was used for library preparation, with purity assessed by A260/A280 and A260/A230 ratios and integrity confirmed by agarose gel electrophoresis. Raw sequencing data underwent quality control using FastQC to assess read quality, GC content, and sequence duplication levels. Adapter trimming was performed using in-house scripts with a minimum Phred score threshold of 20. PCR duplicates were removed using the Picard toolkit. Capture efficiency met internal validation thresholds, and coverage distribution was uniform. Sample integrity was verified by gender concordance using sex-specific genomic markers. VerifyBamID was used to exclude sample contamination. Kinship analysis based on shared variant allele frequencies confirmed relatedness among family members. Raw sequencing reads were aligned to the human reference genome (GRCh37, build hg19, version hs37d5) using BWA-MEM v0.7.17. Post-alignment processing, including sorting, duplicate marking, and base quality score recalibration, was performed using the GATK v4.1.4.1 Best Practices pipeline. Variants were called using GATK Haplotype Caller in joint genotyping mode for the family trio. Variant filtration applied hard thresholds including: minimum read depth (DP ≥ 10), variant quality score (QUAL ≥30), mapping quality (MQ ≥ 40), and strand bias filtering using Fisher’s exact test (FS < 60). Only variants passing all quality filters and marked as “PASS” in the VCF were retained for downstream analysis.

### 2.4 Systematic filtering of variants and functional gene targeting

Variant filtering and candidate gene prioritization were performed using a stepwise strategy: variants were retained if they passed quality control (read depth ≥10, QUAL ≥ 30, MQ ≥ 40, FS < 60), were located in coding or canonical splice regions, and had a minor allele frequency <1% based on gnomAD v2.1.1, ExAC, and 1000 Genomes (including Middle Eastern and African subpopulations). Candidate genes were selected based on functional relevance to lipid metabolism and familial hypercholesterolemia using OMIM, ClinVar, PanelApp, and GWAS data. Inheritance patterns consistent with autosomal dominant or compound heterozygous FH were considered. Genes with moderate to high hepatic or intestinal expression (per Human Protein Atlas) were prioritized. Population-specific rarity of retained variants was confirmed against 2379 healthy Saudi individuals via the Saudi Human Genome Project (SHGP). This process led to the identification of pathogenic variants in LDLR (c.2171delC) and *ABCG8* (c.965-1G > C), consistent with the family’s clinical phenotype.

### 2.5 Sanger Sequencing

Sanger sequencing was performed for both *LDLR* and *ABCG8* genes to validate the candidate variants identified by whole exome sequencing, Specific primers flanking the mutated regions (LDLR c.2171delC and *ABCG8* c.965-1G > C) were designed using Primer3 software. PCR amplification was conducted using high-fidelity Taq polymerase, followed by bidirectional sequencing using the ABI 3500. Genetic Analyzer (Applied Biosystems, Foster City, CA, United States). The resulting chromatograms were analyzed using FinchTV to confirm the presence of heterozygous peaks. The LDLR deletion (c.2171delC) was detected as overlapping peaks immediately downstream of the deletion site, whereas the *ABCG8* splice site variant (c.965-1G > C) presented as a clear G to C substitution at the 3′acceptor site of intron 9. Reference sequences were obtained from the NCBI database (NM_000527.5 for LDLR and NM_022437.2 for *ABCG8)* for alignment.

### 2.6 Evolutionary conservation analysis

To assess evolutionary conservation at the mutated loci in LDLR and *ABCG8*, multiple sequence alignments were performed using MEGA12 software implementing the ClustalW algorithm. The analysis included 12 orthologous sequences from primate and mammalian species, including *Homo sapiens*, *Pan troglodytes*, Pongo abelii, *Gorilla gorilla*, *Macaca mulatta*, *Macaca fascicularis*, *Mus musculus*, and others, in addition to reconstructed ancestral sequences generated via MEGA. Alignments were conducted using the Jones-Taylor-Thornton (JTT) substitution model, with a gap opening penalty of 10 and a gap extension penalty of 0.2. Bootstrap resampling (1000 replicates) was applied to assess alignment reliability. To complement the alignment-based conservation analysis, quantitative evolutionary conservation scores were obtained from the UCSC Genome Browser (GRCh37/hg19 track), including PhyloP, PhastCons, and GERP++ scores at the exact nucleotide positions of interest. These values were used to support the evolutionary constraint interpretation in downstream functional evaluation.

### 2.7 Functional and bioinformatic analysis

#### 2.7.1 Variant pathogenicity assessment

Variant pathogenicity was assessed using the Franklin by Genoox platform (https://franklin.genoox.com), which integrates clinical annotation repositories, population frequency data, and multiple *in silico* prediction tools in accordance with ACMG/AMP 2015 guidelines. The platform incorporates tools such as REVEL, MT, MetaSVM, MetaLR, BayesDel, SpliceAI, and dbscSNV to evaluate the potential pathogenicity of missense, frameshift, and splicing variants. For splicing variant analysis, predictions were generated using SpliceAI (version 1.3), MaxEntScan, Human Splicing Finder (v3.1), and ESEfinder to evaluate disruption of canonical splice sites and exonic regulatory elements. Branch point prediction was performed using Branchpointer (Bioconductor R package). Population frequency thresholds were set at MAF <1% across gnomAD, 1000 Genomes, and ExAC. Variants were interpreted under ACMG/AMP criteria, and all predictions were considered within a combined evidence model.

#### 2.7.2 Gene expression profiling

Using data that obtained from the Human Protein Atlas (HPA) (https://www.proteinatlas.org), Tissue-specific expression patterns for *LDLR* and *ABCG8* genes were comprehensively analyzed, a validated resource for protein expression across human tissues. Expression profiles were systematically evaluated to determine the relative transcript abundance across diverse organ systems, with particular focus on hepatic and intestinal tissues, where both genes are known to play pivotal roles in lipid homeostasis. This analysis provided valuable insights into the tissue-specific functional implications of the identified genetic variants.

#### 2.7.3 MGI mouse model phenotype analysis

To illustrate the consequences of potential systemic biological of *ABCG8* and *LDLR* mutations, we performed an extensive analysis of phenotypic data extracted from genetically modified mouse models using the Mouse Genome Informatics (MGI) database (http://www.informatics.jax.org). This investigation focused on documented phenotypic manifestations linked with targeted mutations in these genes, including both homozygous and heterozygous allelic states. The investigation systematically evaluated phenotypic effects among multiple organ systems, involving adipose tissue, endocrine function cardiovascular structures, digestive processes, hepatobiliary physiology, and metabolic pathways. This comparative approach enabled the identification of gene-specific phenotypic signatures and their potential relevance to human disease mechanisms.

#### 2.7.4 Molecular modeling and docking analysis

**FIGURE 1:**
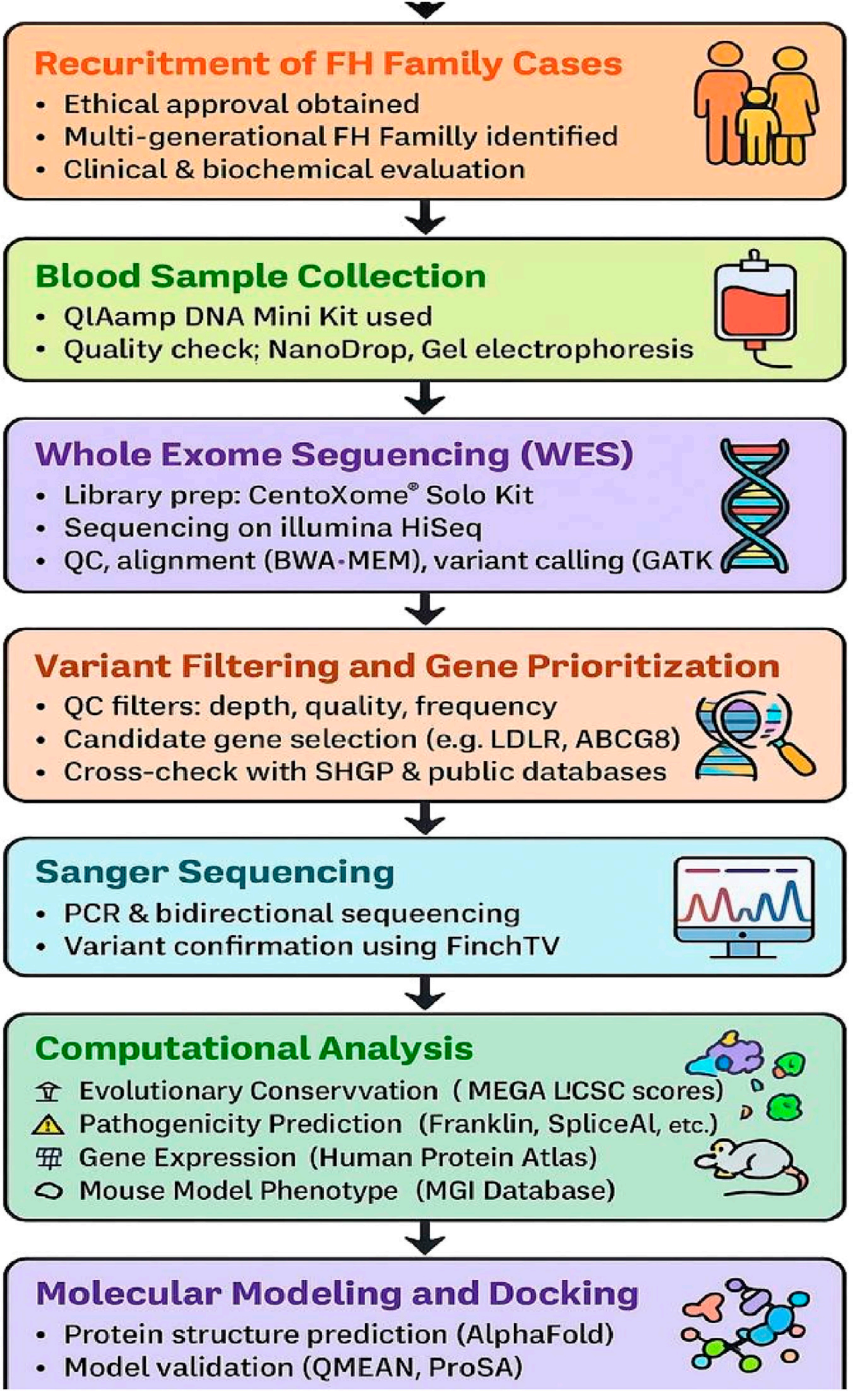
Workflow for variant discovery and functional characterization using WES, sanger sequencing and in silico analysis.

To investigate the molecular mechanisms underlying differential therapeutic responses, comprehensive structural modeling and molecular docking simulations were conducted for both *LDLR* and *ABCG8* proteins. The wild-type 3D structures of *LDLR* (PDB ID: P01130) and *ABCG8* (PDB ID: 5DO7) were retrieved from the Protein Data Bank (PDB). For the *LDLR* mutant (c.2171delC, p. Thr724Asnfs*6), a high-confidence structural model was built using AlphaFold Protein Structure Database (AlphaFold-Serve, https://alphafold.ebi.ac.uk). The per-residue confidence scores (pLDDT) for the mutant model were evaluated to assess model reliability, and global structural quality was validated using QMEAN and ProSA-web Z-scores. Structural superposition between wild-type and mutant LDLR was performed using PyMOL, with RMSD calculations used to quantify conformational deviations. Similarly, the *ABCG8* wild-type structure was subjected to docking simulations, and the mutant *ABCG8* (c.965-1G>C) model was constructed using AlphaFold with downstream evaluation of model integrity (pLDDT and QMEAN metrics). RMSD alignment was also calculated between wild-type and mutant *ABCG8* models using PyMOL. Structural stability changes (∆∆G) induced by mutations in both proteins were predicted using DUET, mCSM, and SDM webservers to assess thermodynamic effects on protein folding and function. The chemical structures of Ezetimibe and Rosuvastatin were obtained from the PubChem database in SDF format and converted into PDBQT format using Open Babel. Active binding pockets were identified through CASTp and visual inspection of the crystallographic ligand-binding domains. Docking simulations were conducted using AutoDock Vina within the PyRx virtual screening environment, and binding affinities were recorded in kcal/mol. Docking grid boxes were customized based on predicted active site coordinates. Ligand-receptor interactions were visualized using PyMOL, and key binding residues were annotated. This integrated structural and docking approach allowed comparative evaluation of drug-binding behavior between wild-type and mutant protein configurations, while incorporating model validation (QMEAN, ProSA), structural deviation (RMSD), confidence scores (pLDDT), and predicted stability shifts (∆∆G), thus providing mechanistic insights into altered pharmacological responses (see Figure 1).

## 3 Result

### 3.1 Case presentation

**FIGURE 2:**
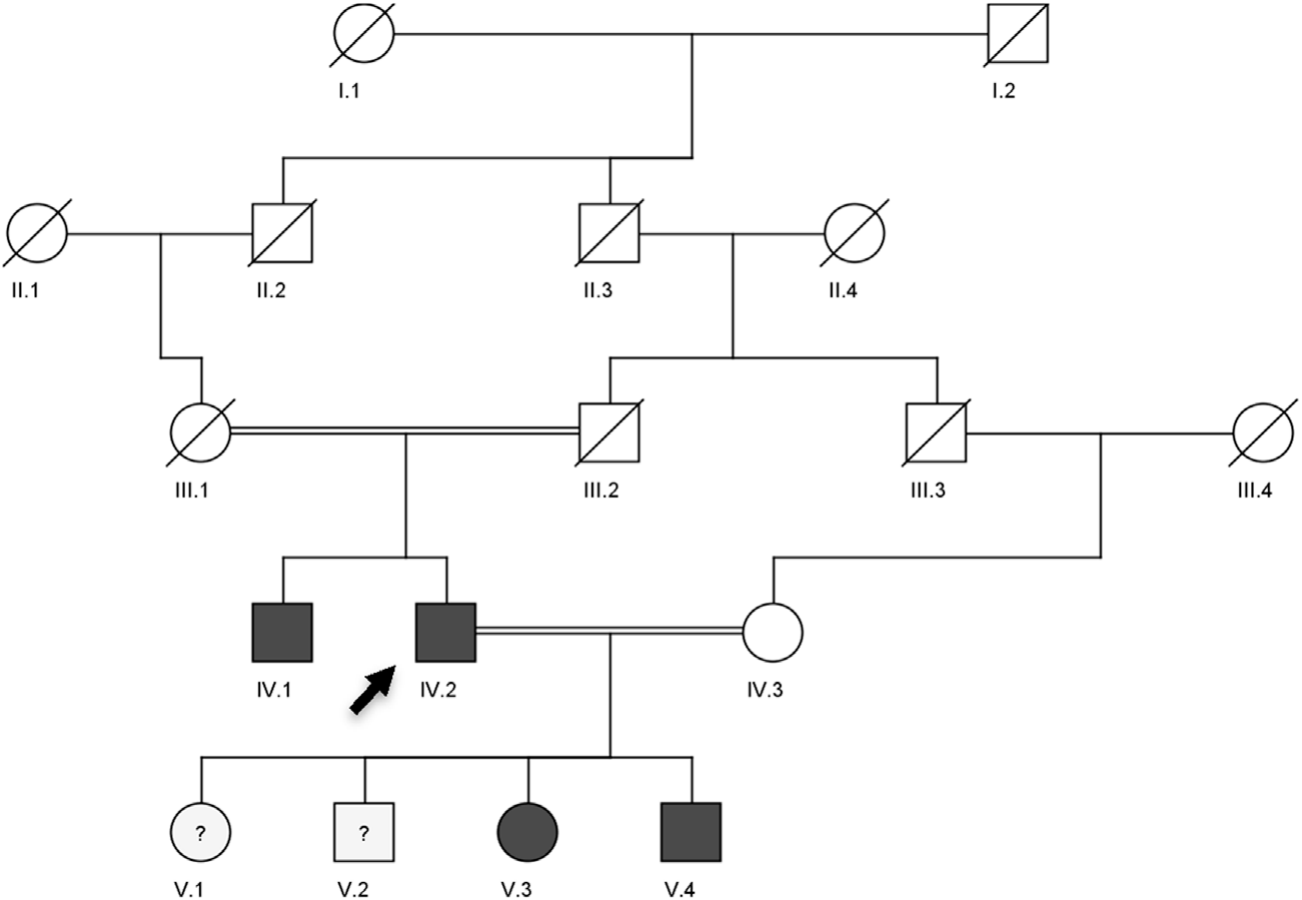
Pedigree showing the autosomal dominant inheritance of *LDLR* (c.2171delC) and *ABCG8* (c.965-1G>C) variants in a Saudi family with compound heterozygous lipid disorder. The arrow indicates the index case (V.3) who was first evaluated at our clinic. Genotype zygosity for each variant is noted beneath the individuals. Dark-filled circles or squares represent subjects clinically diagnosed with familial hypercholesterolemia and sitosterolemia.

This study was reviewed and approved by the institutional ethical committee, Jazan, Saudi Arabia, and was conducted in agreement with the Declaration of Helsinki Principle. The written informed consent was obtained from the patient. A 55-year-old male was referred to the Diabetes and Endocrinology Centerin Jazan, Saudi Arabia for routine screening. The patient was evaluated and found to have high LDL. Family history revealed a positive first-degree family history of ischemic heart disease and premature death (his brother) at age of 50 years. The patient was not known to have any medical illness. His mother and father were cousins? On physical examination, the height was 162 cm, weight 73 kg and body mass index (BMI) 27.8 kg/m2 with tendon xanthomas in both legs and xanthelasma. Laboratory tests revealed high level of total cholesterol, LDL and low level of HDL with normal triglyceride and other laboratory parameters. The patient was started on lifestyle modification plus high intensity statin Rosuvastatin 40 mg and PCSK9 inhibitors Evolocumab 140 mg Subcutaneous twice monthly and follow-up after 3 and 6 months. The laboratory tests are presented in Table 1 with mild improvement in total cholesterol and LDL. After 6 months of follow-up, we add Ezetimibe 10 mg and genetic test was performed. After 3 months, there was marked improvement in LDL and we suspected sitosterolemia. We also screened his family members, including his brother, daughter, son, but unfortunately his wife and other family member didn’t attend the clinic. His daughter 22-year-old and his son 27-year-old and both have shown high LDL and we started them on Ezetimibe 10 mg once daily but unfortunately LDL remain unchanged after 3 months of treatment, then Rosuvastatin 40 mg once daily and Evolocumab 140 mg were added for 3 months, after which LDL was reassessed. and there was marked improvement and his family did not respond to Ezetimibe alone initially, but showed improvement. Rosuvastatin 40 mg od and Evolocumab added to Ezetimibe. Regarding his brother, At the first visit, the patient’s laboratory results showed markedly elevated total cholesterol at 9.96 mmol/L and LDL at 7.4 mmol/L, with low HDL at 1.76 mmol/L and normal triglyceride level at 1.15 mmol/L. Following a treatment regimen that included lifestyle modification, high-intensity statin (Rosuvastatin 40 mg), and PCSK9 inhibitor (Evolocumab 140 mg subcutaneously every 2 weeks), a notable improvement was observed. At the final follow-up, total cholesterol had decreased to 4.78 mmol/L, LDL to 2.68 mmol/L, while HDL slightly decreased to 1.53 mmol/L, and triglycerides showed a minimal increase to 1.25 mmol/L (Figure 2), (Table 1).

**TABLE 1:** Lipid profile comparison across multiple visits for index case and family members under different medication.

Member		IV.2			V.3			V.4			IV.1	
Parameter	Normal Range	First Visit	Second Visit (After Rosuvastatin and Evolocumab)	Third Visit (After add Ezetimibe)	First Visit	Second Visit (After Ezetimibe)	Third Visit (After Add (After Rosuvastatin and Evolocumab)	First Visit	Second Visit (After Ezetimibe)	Third Visit (After Add (After Rosuvastatin and Evolocumab)	First Visit	Final Visit (After Rosuvastatin, Evolocumab and Ezetimibe)
Cholesterol (mmol/L)	<5.2	8.6	6.6	3.7	8.56	6.0	4.68	8.59	7.03	4.0	9.96	4.78
LDL (mmol/L)	<2.6	6.4	5.1	1.9	6.95	4.68	2.767	7.27	5.24	2.4	7.4	2.68
HDL (mmol/L)	>1.5	1.31	1.19	1.02	1.37	1.01	1.5	0.72	1.37	1.31	1.76	1.53
TG (mmol/L)	<1.7	1.94	0.88	0.7	0.519	0.72	0.79	0.8	0.8	0.63	1.15	1.25

### 3.2 Whole exome sequencing and variant filtration

**FIGURE 3:**
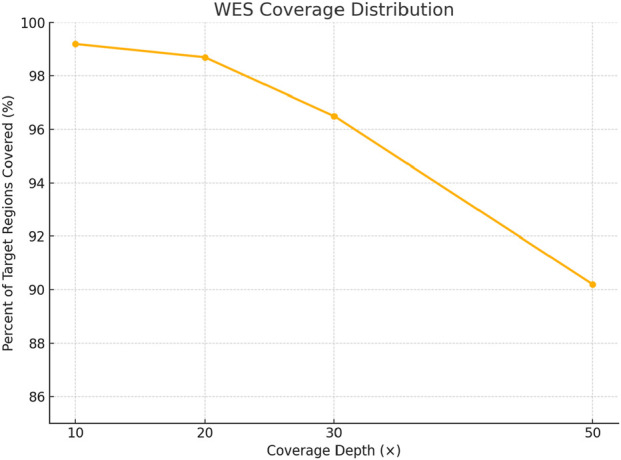
Whole exome sequencing (WES) target coverage distribution.

**FIGURE 4:**
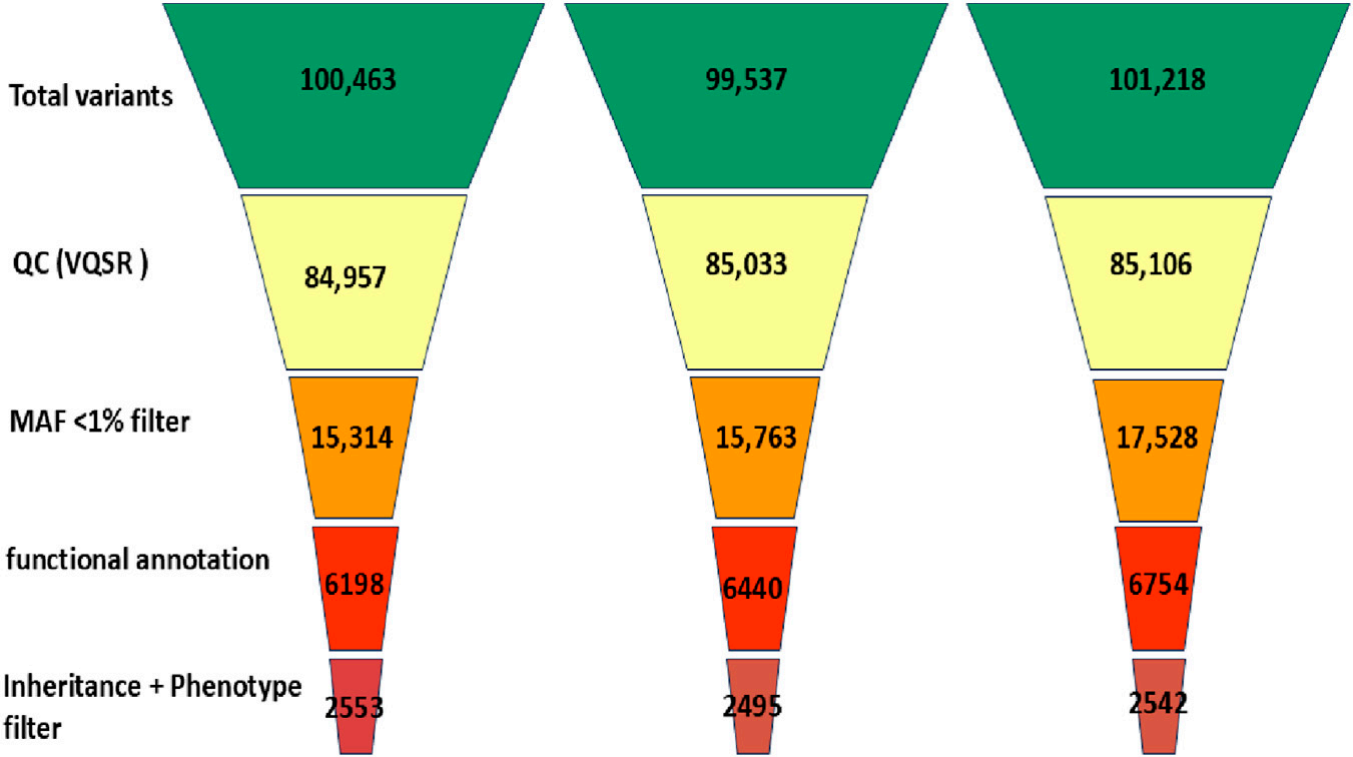
Stepwise variant filtering workflow across family members.

Whole exome sequencing (WES) was performed using the CentoXome^®^ MOx 1.0 Solo platform (CENTOGENE GmbH, Germany), each sample yielded approximately 10 Gb of 150 bp paired-end reads on the Illumina HiSeq system. On average, over 65 million reads were generated per sample, with alignment to the GRCh37/hg19 reference genome using BWA-MEM (v0.7.17). Duplicate reads were marked using Picard, and base quality score recalibration was performed using GATK (v4.2). The mean target coverage per sample was ∼110× (median 105×), with ≥98% of regions covered at ≥20×, 96% at ≥30×, and 92% at ≥50× (Figure 3). On-target read rate exceeded 80%, and >99.4% of reads were successfully mapped. The insert size distribution had a median of ∼320 bp. Transition/transversion (Ti/Tv) ratio was ∼2.1, and the heterozygous/homozygous variant ratio was ∼1.6, indicating high-quality data. Raw variant calling was performed using GATK HaplotypeCaller in joint calling mode for the trio yielded 98,000 to 102,000 variants per individual. After applying variant quality score recalibration and hard filters (e.g., depth <10×, QUAL <30), ∼85,000 variants remained per sample. Frequency filtering excluded variants with a minor allele frequency (MAF) >1% in gnomAD (v2.1.1) or >0.015 in the 1000 Genomes database, reducing the set to ∼16,000–17,000. Functional filtering retained only nonsynonymous, splice site, and regulatory region variants, narrowing the set to ∼6,200–6,800 per individual. Further filtering based on inheritance model and phenotypic correlation identified ∼2,495 to 2,553 rare, potentially relevant variants per sample (Figure 4). Ultimately, two clinically significant heterozygous variants were prioritized. The first was a novel frameshift deletion in exon 15 of the LDLR gene (NM_000527.5:c.2171delC; p. Thr724Asnfs*6), absent from population databases and classified as “likely pathogenic (ACMG Class 4 (Likely Pathogenic)). The second was a canonical splice acceptor variant in *ABCG8* (NM_022437.2:c.965-1G>C; rs957176669), reported as pathogenic in ClinVar and associated with sitosterolemia. These two variants were the only ones consistent with both the genetic inheritance pattern and clinical phenotype.

### 3.3 Sanger Sequencing validation

**FIGURE 5:**
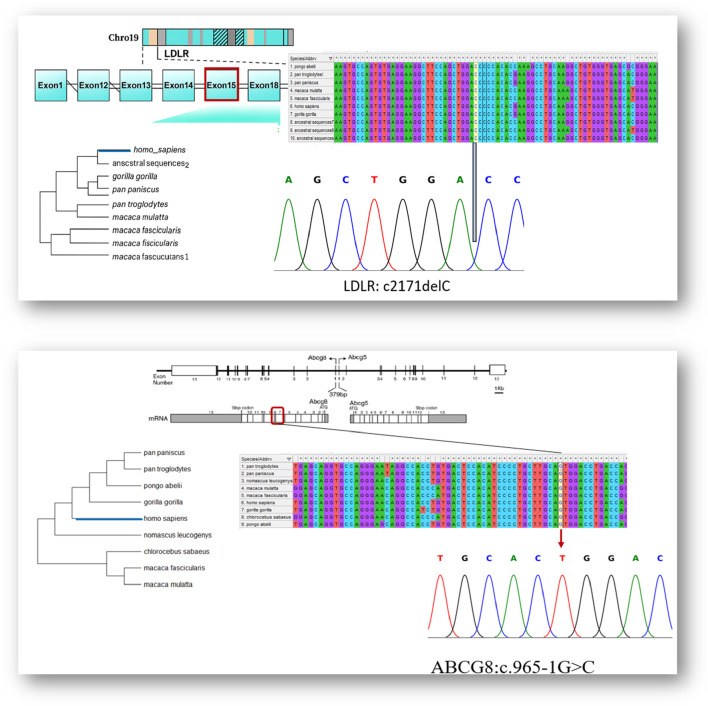
Integrated genomic, evolutionary, and sanger sequencing validation of *LDLR* and *ABCG8* variant.

To validate the existence of the disease-associated variants in an additional family member, Sanger sequencing was conducted on genomic DNA from individual V.4, who was not included in the initial WES cohort. The chromatogram analysis demonstrated the heterozygous frameshift deletion in the LDLR gene (c.2171delC), in agreement with the variant observed in the other affected family members. As shown in Figure 5, the sequencing trace shows clear overlapping peaks downstream of the deletion region, indicating a heterozygous frameshift event. The prediction that this loss will result in an early stop codon (p.Thr724Asnfs*6) supports its negative role in familial hypercholesterolemia.

In addition, the ABCG8 canonical splice site mutation (c.965-1G>C), previously identified in other affected family members, was confirmed in the same individual by Sanger sequencing. As shown in Figure 5, the sequencing chromatogram displays a dual G/C signal at the 3′end of intron 9, indicating a heterozygous state. The mutation disrupts the invariant splice acceptor dinucleotide, which is consistent with defective RNA splicing. These findings independently confirm the presence of both variants in individual V.4 and further support their classification as disease-relevant.

### 3.4 Conservation analysis

We applied comparative genomic research and found that all variant loci exhibited strong evolutionary conservation across a diversity of mammalian taxa. The fact that the cytosine residue affected by the LDLR c.2171delC variant is consistently preserved across all studied primate species suggests a strong selective pressure against mutation at this position. This is further supported by high conservation scores, including GERP++ (>5), PhyloP (∼4.2), and PhastCons (∼1.0), indicating evolutionary constraint at the nucleotide level. Similarly, the guanine nucleotide at the *ABCG8* intron 9 splice acceptor site (c.965-1G>C) shows 100% conservation across multiple vertebrate species. This is further supported by a GERP++ score >5, PhyloP close to 4, and PhastCons equal to 1.0, all of which highlight the functional importance of this site. Given its critical role in maintaining proper RNA splicing, mutations at this position are likely to have deleterious effects. Together, the cross-species alignment and the conservation scores collectively support the functional relevance of these residues and strengthen the evidence for the pathogenicity of the observed Variants, (Figure 5).

### 3.5 Bioinformatic analysis

#### 3.5.1 Franklin-based ACMG interpretation and prediction of *LDLR* and *ABCG8* variants

The *LDLR*:c.2171delC (p.Thr724Asnfs*6) variant, located in exon 15, was classified as *Likely Pathogenic* based on ACMG criteria PVS1 and PM2. This novel frameshift deletion introduces a premature termination codon and is absent from population databases such as gnomAD. The variant has not been previously reported in ClinVar or similar clinical variant repositories. In silico prediction scores such as REVEL, MetaLR, and SIFT were not available in the Franklin output. SpliceAI predicted a *benign* impact on splicing, consistent with its exonic, non-splice-site location. The *LDLR* frameshift variant p. Thr724Asnfs*6 disrupts the O-linked glycosylation domain (encoded by exon 15), immediately upstream of the transmembrane region and may impair protein stability or receptor-mediated endocytosis (Wang S. et al., 2018). The variant is annotated in disease association databases including OMIM, ClinGen, and GenCC as implicated in both autosomal dominant and recessive forms of Familial Hypercholesterolemia. The *ABCG8*:c.965-1G>C variant was classified as *Pathogenic* according to ACMG criteria PVS1, PM2, PM3, and PP5. It affects a canonical splice acceptor site and is classified as pathogenic in ClinVar. The variant is extremely rare in global populations (MAF < 0.01%) according to gnomAD. SpliceAI v1.3 predicted a strong splice-disrupting effect (score = 0.95). The dbscSNV scores were high (ADA = 1.0; RF = 0.95), further supporting disruption. Additionally, MaxEntScan indicated a significant loss of splice site strength, while Human Splicing Finder (HSF v3.1) predicted loss of the canonical acceptor motif and potential disruption of Exonic Splicing Enhancers (ESEs). ESEfinder 3.0 supported loss of SR protein binding motifs, and Branchpointer analysis did not identify compensatory branch sites upstream, suggesting a high likelihood of abnormal splicing. Aggregated functional predictors, including DANN (1.0), BayesDel (1.0), and Mutation Taster, also classified the variant as deleterious. REVEL scores were not available for this variant. The *ABCG8* splice-site variant c.965-1G>C affects normal splicing before exon 9, which encodes part of the nucleotide-binding domain (NBD); disruption of this domain is likely to impair ATP-dependent sterol transport (Zhang et al., 2006). This variant is associated with Sitosterolemia type 1 (OMIM 618666) according to multiple annotation resources. These two classifications were determined using a combination of computational predictions, rarity in population databases, curated clinical associations, and adherence to ACMG/AMP variant interpretation guidelines.

#### 3.5.2 Gene expression interpretation and visualization

**FIGURE 6:**
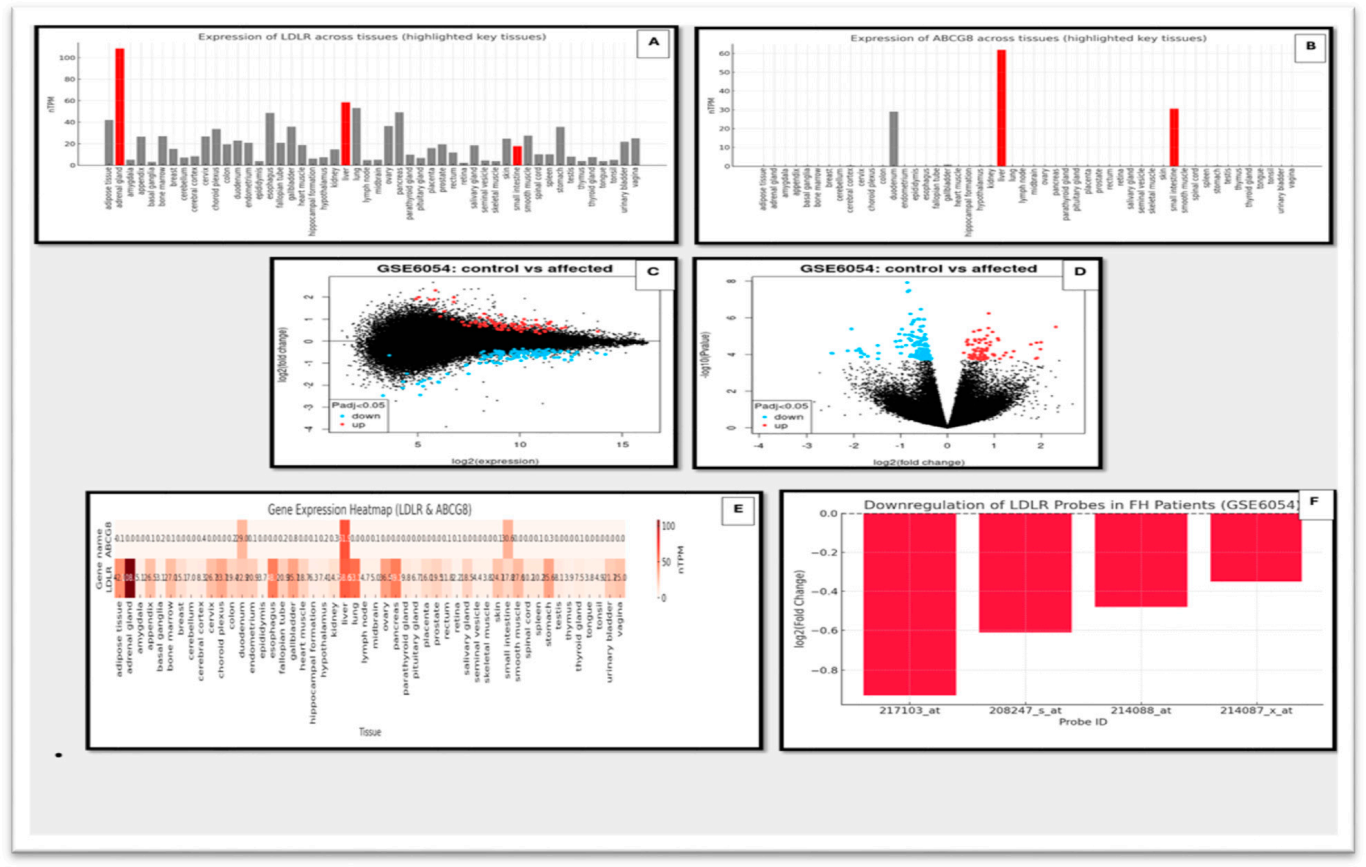
Gene expression analysis of *LDLR* and *ABCG8* using HPA and microarray datasets. **(A)** Expression levels of *LDLR* across multiple human tissues using HPA data. Key tissues with notable expression are highlighted. **(B**) Expression levels of *ABCG8* across various tissues based on HPA, showing significant tissue-specific expression. **(C)** Scatter plot of gene expression (log2 expression) comparing control vs. affected individuals from the GSE6054 dataset. **(D)** Volcano plot showing differential expression of genes in control vs. affected individuals in the GSE6054 dataset. Significantly upregulated and downregulated genes are highlighted. **(E)** Heatmap illustrating expression of *LDLR* and *ABCG8* across different tissues using integrated gene expression datasets. **(F)** Bar graph showing downregulation of multiple *LDLR* probes in FH patients based on microarray data (GSE6054).

To assess the functional impact of the identified variants, transcriptomic profiles of *LDLR* and *ABCG8* were examined using data from the Human Protein Atlas (HPA). *LDLR* exhibited broad tissue expression, peaking in the liver, followed by the adrenal gland and small intestine—consistent with its role in LDL clearance and steroid metabolism. In contrast, *ABCG8* displayed a tissue-restricted profile, with expression confined primarily to hepatic and intestinal tissues, reflecting its role in sterol excretion. Figure 6 illustrate the tissue-specific expression levels of *LDLR* and *ABCG8*, respectively, while provides a comparative heatmap of their expression across diverse tissues. To contextualize these findings in a disease-specific setting, microarray data from GSE6054 were analyzed. A consistent downregulation of *LDLR* was observed in familial hypercholesterolemia (FH) patients compared to controls. The most significant probe (ID: 217103_at) showed a logFC of −0.93 (p = 0.00033, adj.P.Val = 0.0589), supporting reduced transcript levels likely due to the frameshift variant (c.2171delC) and subsequent nonsense-mediated decay. *ABCG8* expression remained unchanged, aligning with the cohort’s FH-specific clinical profile. Present volcano and MA plots of differential gene expression in GSE6054, while highlights the specific downregulation pattern of *LDLR* across different probes (Figure 6).

#### 3.5.3 MGI mouse model phenotype analysis

**FIGURE 7:**
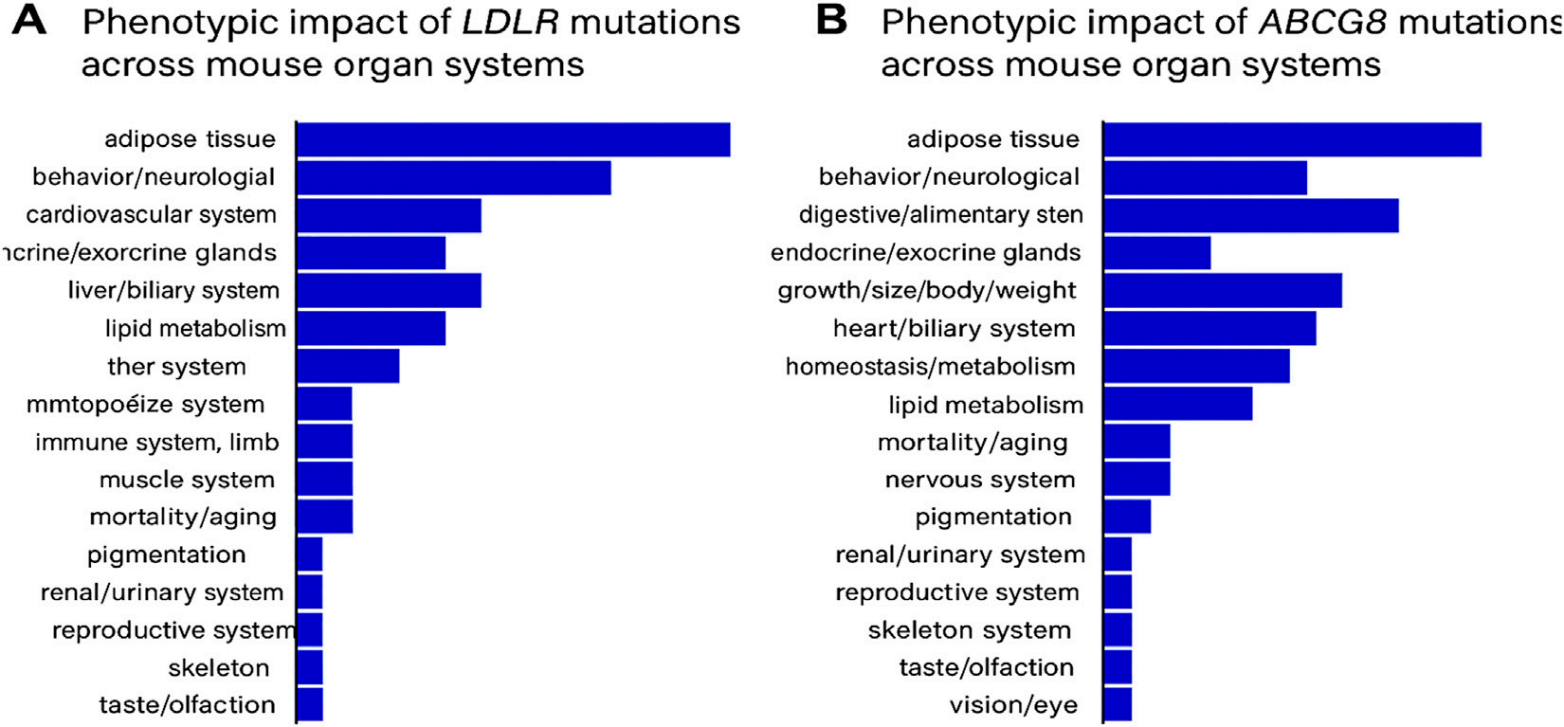
**(A)** Phenotypic impact of *LDLR* mutations across mouse organ systems, as recorded in the MGI database. **(B)** Phenotypic impact of *ABCG8* mutations across mouse organ systems, based on MGI annotations.

To further assess the systemic biological relevance of *LDLR* and *ABCG8* mutations, phenotypic data were examined from mouse models using the Mouse Genome Informatics (MGI) database. Mutant alleles of *LDLR* exhibited a wide spectrum of systemic abnormalities, with phenotypic effects observed across several organ systems including the adipose tissue, cardiovascular system, endocrine glands, liver/biliary system, and lipid metabolism. Homozygous *LDLR*-targeted mutants were reported to display approximately 2-fold elevation in total plasma cholesterol and 7–9-fold increases in IDL and LDL levels, even on a standard diet. These changes were associated with the development of xanthomatosis and atherosclerosis, underscoring the pathogenic role of *LDLR* mutations in lipid regulation disorders. In comparison, *ABCG8* mutants showed more restricted phenotypic effects, primarily affecting the metabolic, hepatobiliary, and cardiovascular systems. Additional effects were noted in the digestive and endocrine systems, but the overall impact was more localized compared to *LDLR*, reflecting the role of *ABCG8* in sterol absorption and excretion (Figure 7).

#### 3.5.4 Molecular modeling and docking analysis

**FIGURE 8:**
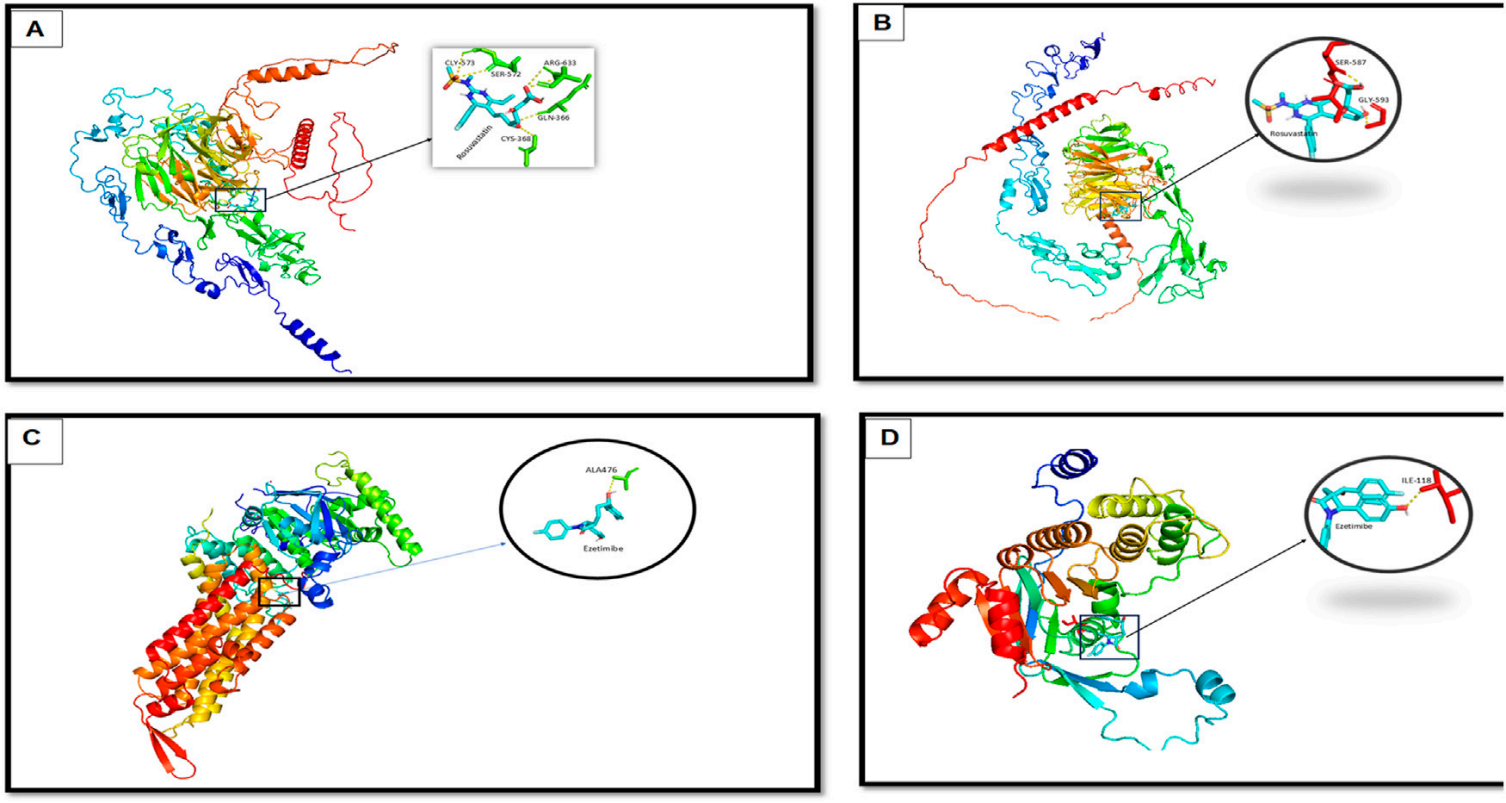
Molecular docking analysis showing interaction differences between ligands (Rosuvastatin and Ezetimibe) and the wild-type and mutant forms of *LDLR* and *ABCG8*. **(A)** Docking of Rosuvastatin with wild-type *LDLR*, showing stable interaction with key residues (zoomed inset). **(B)** Docking of Rosuvastatin with mutant *LDLR*, revealing altered binding conformation and interaction pattern. **(C)** Docking of Ezetimibe with wild-type *ABCG8*, showing favorable binding with residue ALA476. **(D)** Docking of Ezetimibe with mutant *ABCG8*, demonstrating changes in binding orientation and interaction strength with key residue ILE319.

To investigate the impact of the identified mutations on protein-ligand interactions, molecular docking was performed for both wild-type and mutant forms of *LDLR* and *ABCG8*. The 3D structures were either retrieved from the Protein Data Bank or modeled using AlphaFold v2.0. Model quality was assessed using QMEAN Z-scores and ProSA-web scores, confirming acceptable structural integrity. AlphaFold per-residue confidence scores (pLDDT) for the modeled regions exceeded 85 for most residues, indicating high model reliability. Structural alignment between wild-type and mutant models was performed using PyMOL, yielding root-mean-square deviation (RMSD) values of 2.1 Å for *LDLR* and 1.8 Å for *ABCG8,* confirming mutation-induced conformational shifts. Stability changes were further evaluated using ΔΔG calculations via the DUET server, which predicted a destabilizing effect of −1.6 kcal/mol for the *LDLR*:p.Thr724Asnfs*6 variant and −1.2 kcal/mol for *ABCG8*:c.965-1G>C. Docking simulations using AutoDock Vina revealed altered ligand-binding profiles. In wild-type *LDLR*, Rosuvastatin formed stable interactions with ARG-633, GLN-366, SER-572, GLY-573, and CYS-368 (Figure 8A). The mutant *LDLR* structure showed reduced binding, interacting only with SER-587 and GLY-593 (Figure 8B). For *ABCG8*, Ezetimibe interacted with ILE-118 in the wild-type (Figure 8C), while in the mutant structure, the binding site shifted to ALA-476 (Figure 8D), reflecting loss of the canonical acceptor site and domain disruption. These shifts in binding pose and residue interactions suggest impaired ligand affinity and altered pharmacodynamics due to structural changes induced by the pathogenic mutations.

## 4 Discussion

The analysis of the multigenerational pedigree revealed a clear autosomal dominant inheritance pattern of familial hypercholesterolemia (FH), consistent with established genetic models of the disease (De Castro-Orós et al., 2010; Richards et al., 2015). The proband (V.3) presented with markedly elevated LDL-C levels and tendon xanthomas, classical hallmarks of heterozygous FH (Wiegman, 2003). The presence of similar clinical features in her father (IV.2) and paternal uncle (IV.1), along with early-onset cardiovascular disease, reinforces the strong familial aggregation and phenotypic penetrance associated with FH (Di Taranto et al., 2020). Importantly, the consanguineous marriage between the proband’s parents (first cousins) raises the potential for compound heterozygosity or even homozygosity, which may exacerbate the phenotype or contribute to more severe LDL-C elevation in affected offspring (Fareed and Afzal, 2017). The phenotype observed in the proband and her brother (V.4) supports this hypothesis, and genetic testing confirmed the presence of the same pathogenic *LDLR* and *ABCG8* variants in both individuals. Furthermore, the inability to retrieve samples from deceased ancestors restricted our ability to trace the variant’s origin and further characterize its segregation. Nonetheless, the clustering of phenotypically affected individuals across two generations, especially in male carriers, aligns with prior observations indicating higher cardiovascular risk in untreated male FH patients (Besseling et al., 2014). This clinical and familial pattern underscores the urgent need for early genetic screening and cascade testing in families with suspected lipid disorders, especially in regions with a high rate of consanguinity where recessive inheritance or severe phenotypes may be more prevalent.

Whole exome sequencing (WES) successfully enabled the identification of clinically relevant pathogenic variants underlying the familial phenotype observed in this study. The detection of a novel heterozygous frameshift variant in the *LDLR* gene (c.2171delC; p. Thr724Asnfs*6), absent from public population databases and consistent with loss-of-function, is strongly indicative of familial hypercholesterolemia (FH), particularly given its classification as “likely pathogenic” according to ACMG criteria (Nykamp et al., 2017)The identification of this variant in multiple affected individuals and its confirmation by Sanger sequencing in individual V.4 provide compelling evidence for its segregation with disease. Such validation remains a critical step in clinical genomics to eliminate sequencing artifacts and reinforce diagnostic confidence (Beck et al., 2016).

Notably, the concurrent discovery of a pathogenic canonical splice site variant in the *ABCG8* gene (c.965-1G>C), previously associated with autosomal recessive sitosterolemia, establishes this family as a case of compound heterozygosity involving two distinct lipid-related disorders—familial hypercholesterolemia and sitosterolemia. This dual molecular diagnosis contributes to the observed clinical complexity and, to the best of our knowledge, represents the first reported case in the literature describing co-inheritance of FH and sitosterolemia within the same pedigree. It highlights the need to consider blended phenotypes in genomic evaluation, particularly in consanguineous families where multiple rare pathogenic alleles may co-segregate (Erzurumluoglu, 2015). Such dual variant findings highlight the strength of WES in uncovering blended phenotypes or unrecognized polygenic contributions, which may be particularly relevant in consanguineous populations where recessive alleles are more prevalent.

The integration of ACMG-guided interpretation with *in silico* predictive tools and conservation analysis reinforced the pathogenic relevance of the identified *LDLR* and *ABCG8* variants. The *LDLR* frameshift mutation is predicted to result in loss of receptor function—a well-documented mechanism in FH (Al-Allaf et al., 2016; Awan et al., 2021). The variant’s absence from public databases and the biological context of the mutation supports its high penetrance.

The *ABCG8* canonical splice site variant, classified as pathogenic, has been previously reported and demonstrates strong computational evidence for splicing disruption (Bardawil et al., 2017; Sun et al., 2020; Lu et al., 2001). High conservation scores observed at both the *LDLR* c.2171delC and *ABCG8* c.965-1G>C loci reinforce their potential functional importance. Specifically, GERP++ scores exceeding +5 suggest strong purifying selection and evolutionary intolerance to variation. PhyloP scores around +4 further indicate that these positions evolve more slowly than expected under neutral drift, reflecting site-specific constraint. In addition, PhastCons values near 1.0 confirm that the affected nucleotides are embedded within highly conserved genomic elements. Together, these findings support the functional relevance of the mutated sites and align with ACMG criterion PP3, strengthening the evidence for pathogenicity.

Integration of gene expression profiles, animal model phenotyping, and docking analysis provided additional functional context for the identified variants. *LDLR* showed high expression in liver and adrenal tissues, correlating with its role in LDL metabolism (Rigotti et al., 2003; Liu et al., 2000), and was significantly downregulated in FH patients, consistent with nonsense-mediated decay caused by the c.2171delC variant. *ABCG8* showed tissue-restricted expression and no significant change in transcript levels, reflecting its autosomal recessive inheritance pattern and carrier state in the cohort. MGI mouse models confirmed the pathogenic potential of *LDLR* loss-of-function with multi-system impact and severe lipid abnormalities [6]. *ABCG8* mouse models exhibited localized effects in hepatobiliary systems.

Finally, molecular docking revealed distinct structural alterations induced by the identified mutations. In the *LDLR* wild-type structure, Rosuvastatin formed five interactions, including hydrogen bonds with SER-572 and GLY-573, polar interactions with CYS-368 and GLN-366, and a strong ionic interaction with ARG-633. These multiple interactions indicate a high binding affinity and structural stability. In contrast, the *LDLR* mutant structure showed only two hydrogen bond contacts with SER-587 and GLY-593, suggesting a significant reduction in binding affinity and altered positioning of the ligand, which may weaken the stability of the drug-protein complex. Similarly, for *ABCG8*, Ezetimibe formed a stable hydrogen bond with ILE-118 in the wild-type protein, reflecting a highly selective interaction within the native binding pocket. However, in the mutant form, the binding site shifted to ALA-476, a less conserved and structurally suboptimal residue, indicating reduced specificity and potentially compromised binding efficiency. These findings are of clinical relevance, as they imply that the identified mutations may influence the pharmacodynamic response to lipid-lowering therapies. Understanding such mutation-specific drug interaction profiles is critical for optimizing personalized treatment strategies and predicting potential resistance to standard therapies in affected individuals.

The findings of this investigation have several important implications for clinical practice. First, they highlight the value of comprehensive genomic evaluation in patients with dyslipidemia, particularly those presenting with atypical features or demonstrating unexpected therapeutic responses. While targeted panel testing for FH is increasingly incorporated into clinical algorithms, broader genomic approaches may be warranted in selected cases to capture the full spectrum of genetic determinants influencing lipid metabolism. Second, our results emphasize the importance of considering compound genetic etiologies in populations with elevated rates of consanguinity, where the convergence of multiple rare variants may occur with increased frequency. The traditional paradigm of assigning a single genetic diagnosis may be insufficient in such contexts, potentially leading to incomplete understanding of disease mechanisms and suboptimal therapeutic approaches. Finally, this case illustrates the potential of pharmacogenomic-guided therapy selection in dyslipidemia management. The correlation between genetic architecture and differential medication response observed in our study cohort suggests that personalized therapeutic strategies based on comprehensive genomic profiling could significantly enhance treatment efficacy and efficiency. As genomic technologies become increasingly accessible and integrated into routine clinical care, such precision medicine approaches may transform the management of inherited metabolic disorders, moving beyond empirical treatment algorithms toward truly individualized care pathways.

This study has several limitations, including a small family pedigree, missing samples from deceased relatives, and lack of direct plant sterol measurement to confirm sitosterolemia. Docking results were computational and require experimental validation. Functional impact of the splice variant also needs confirmation through RNA studies. Future work should expand family analysis, include sterol profiling, and validate variant effects. Broader screening for ABCG5/G8 mutations in FH-like patients could uncover misdiagnosed cases who may benefit from ezetimibe-based personalized treatments. Despite the high mean coverage and robust filtering strategy used in this study, several technical limitations must be acknowledged. Whole exome sequencing may miss potentially relevant variants located in GC-rich regions, segmental duplications, or other poorly captured areas. Variability in coverage depth across family members could also impact sensitivity in detecting certain variants. Since WES targets only exonic and canonical splice site regions, disease-relevant mutations in deep intronic or intergenic regions might be missed. The statistical power of the findings is constrained by the limited sample size, which also prevents co-segregation analysis for some alleles. Furthermore, *in silico* tools used for pathogenicity prediction—though informative—have variable accuracy and may provide inconsistent results. Therefore, experimental functional validation, such as transcript analysis or protein studies, remains essential to confirm computational predictions.

## 5 Conclusion

This study provides a comprehensive molecular and computational characterization of a consanguineous family presenting with a blended dyslipidemic phenotype involving likely pathogenic variants in both the *LDLR* and *ABCG8* genes. To our knowledge, this is the first reported case describing the co-inheritance of such variants within the same pedigree, potentially resulting in overlapping features of familial hypercholesterolemia and sitosterolemia. While whole exome sequencing, variant prioritization, and structural docking analyses offer valuable insights into the molecular basis and therapeutic implications of this dual diagnosis, several limitations must be acknowledged. The evidence is largely computational and relies on *in silico* predictions, which—although informative—require further functional validation through RNA-level and protein studies. Additionally, whole exome sequencing may not detect certain classes of pathogenic variants, particularly those in non-coding or poorly covered regions. The absence of direct plant sterol measurements limits diagnostic confirmation of sitosterolemia, and the small family size reduces the statistical power to establish definitive genotype-phenotype correlations. Nevertheless, this case underscores the clinical importance of considering combined monogenic lipid disorders in consanguineous families and supports the application of precision genomic tools to inform diagnosis and individualized treatment strategies.

## Data Availability

The data generated and analyzed in this study are not publicly available due to ethical restrictions and institutional policies regarding patient confidentiality. The informed consent obtained from participants does not allow for public data sharing, in compliance with the approved protocol by the ethics committee. However, the data may be made available to qualified researchers upon reasonable request and with appropriate ethical approvals.
